# A Review on the Recent Developments and Perspectives of Copper-Based Nanoparticles for the Electrochemical Reduction of NO_x_ and CO_x_

**DOI:** 10.3390/nano16191253

**Published:** 2026-10-03

**Authors:** M. R. Rosales-Martínez, M. A. Hernández-Pérez, Patricia Maldonado-Altamirano, Claudia Camacho-Zuñiga, E. Ramírez-Meneses, A. Manzo-Robledo

**Affiliations:** 1 Departamento de Ingeniería Química, Industrial y de Alimentos, Universidad Iberoamericana, Prolongación Paseo de la Reforma 880, Lomas de Santa Fe, Mexico City 01219, Mexico; a2078193@correo.uia.mx; 2 Instituto Politécnico Nacional, Departamento de Ingeniería en Metalurgia y Materiales, Escuela Superior de Ingeniería Química e Industrias Extractivas, Unidad Profesional Adolfo López Mateos, San Pedro Zacatenco, Mexico City 07738, Mexico; mahernandezpe@ipn.mx; 3 Escuela Superior de Física y Matemáticas, Instituto Politécnico Nacional, Edificio 9, Unidad Profesional Adolfo López Mateos, San Pedro Zacatenco, Mexico City 07738, Mexico; pmaldonado@upn.mx; 4 Institute for the Future of Education, School of Engineering and Sciences, Tecnologico de Monterrey, Av. Eugenio Garza Sada 2501 Sur, Col. Tecnologico, Monterrey 64700, Mexico; claudia.camacho@tec.mx; 5 Instituto Politécnico Nacional, Departamento de Ingeniería Química SEPI, Escuela Superior de Ingeniería Química e Industrias Extractivas, Unidad Profesional Adolfo López Mateos, Mexico City 07738, Mexico

**Keywords:** NO_x_ and CO_x_ electroreduction, synthesis methods, copper-based materials, nanoparticles

## Abstract

Anthropogenic emissions of carbon oxides (CO_x_) and nitrogen oxides (NO_x_) arising from industrial activities contribute to environmental and health problems, motivating the development of effective strategies for their mitigation and valorization. Among the available approaches, electrochemical reduction offers the additional possibility of converting CO_x_ and NO_x_ species into value-added products that may serve as chemical feedstocks or energy carriers, thereby contributing to carbon and nitrogen resource recovery. However, the practical development of these processes requires catalysts with high activity, selectivity, and stability, supported by synthesis strategies capable of controlling their active structures. Several metals, including Ir, Pt, Rh, Ru, Pd, Ni, Ag, Au, and Cu, have been investigated for the electrochemical reduction of CO_x_ and NO_x_ in aqueous media. Among them, Cu is particularly attractive because of its relatively high abundance, tunable oxidation states, and ability to catalyze both reaction families. This review examines Cu-based nanomaterials prepared through different synthesis strategies and analyzes how composition, oxidation state, morphology, exposed facets, defects, alloying, and interfacial structure influence CO_x_ and NO_x_ electroreduction. By comparing both reaction families, the review identifies shared structure–active-site–performance relationships together with reaction-specific requirements for intermediate stabilization and product formation. Particular attention is given to in situ and operando evidence showing that Cu-based catalysts may reconstruct under electrochemical conditions, so that the catalytically relevant material can differ from the as-synthesized structure. These insights support catalyst optimization with mechanistically informed design of dynamic Cu-based interfaces for selective CO_x_ and NO_x_ electroreduction.

## 1. Introduction

Anthropogenic releases of carbon oxides and oxidized nitrogen species affect atmospheric and aquatic environments through different pathways. Fossil fuel combustion and industrial activities release CO_2_, CO, and nitrogen oxides, principally NO and NO_2_ [1,2,3]. CO_2_ accumulation contributes to climate change and ocean acidification [4], whereas CO toxicity and the participation of NO_x_ in secondary pollutant formation raise concerns about air quality and human health [3]. In aquatic environments, nitrate and nitrite contamination associated with agricultural runoff and wastewater create additional challenges for water quality and nitrogen management [5]. Although these species differ in their sources, environmental behavior, and effects, their mitigation presents a shared challenge: treating residual carbon- and nitrogen-containing streams while limiting further environmental burdens [1,6,7,8]. Figure 1 summarizes the atmospheric emission sources and associated impacts of CO_x_ and NO_x_, together with their potential conversion into useful products.

Alongside emission prevention and pollution control, electrochemical reduction offers opportunities to recover value from these streams by converting CO_2_ and CO into chemicals and fuels [9], and oxidized nitrogen species into products such as ammonia [10,11]. When coupled with low-carbon electricity, these transformations may connect environmental treatment with resource recovery [12,13]. Their practical implementation, however, requires control over competing reaction pathways, energy consumption, and catalyst degradation [12,14]. Developing catalysts that combine activity, product selectivity, and stability is therefore central to realizing this potential.

Copper-based nanomaterials provide a versatile platform for investigating both reaction families because their composition, surface structure, and electronic properties can be adjusted through synthesis and subsequent modification. Their performance depends on the interplay among oxidation state, morphology, exposed facets, defects, alloying, doping, and support interactions [13,15]. Moreover, the initial material may undergo reconstruction during electrolysis, complicating the identification of catalytically relevant sites [14,15]. Comparing carbon and nitrogen electroreduction is therefore useful for examining how related catalyst-design variables influence distinct transformations. Both involve adsorbed intermediates, multiple electron-transfer steps, and competition with hydrogen evolution, while their different intermediates and desired products impose specific requirements on the active sites [12]. Figure 2 summarizes this conceptual framework, linking catalyst design, structure and active sites, and catalytic performance, with characterization and theoretical analysis supporting the interpretation of these relationships.

Previous reviews have examined complementary aspects of Cu-based electrocatalysis. Huang et al. analyzed the pathways toward CO and HCOOH [16]. Li et al. [15] reviewed CO_2_ electroreduction from copper nanoparticles to single atoms, relating catalyst structure and coordination to product selectivity. Ullah et al. [13] discussed Cu-based nitrate-to-ammonia electrocatalysis, including structural regulation, support effects, and advanced characterization. Jia et al. [12] addressed CO_2_, N_2_, and nitrate electroreduction through models of cooperation in multicomponent catalysts. These studies provide a basis for comparing catalyst-design strategies across carbon and nitrogen electroreduction. Building on this foundation, the present review examines how synthesis and structural evolution shape the performance of Cu-based nanomaterials in both reaction families, emphasizing the evidence used to identify active sites and distinguish shared design principles from reaction-specific requirements.

Accordingly, this review examines Cu-based nanomaterials for the electrochemical reduction of CO_2_ and CO and of oxidized nitrogen species, particularly nitrate, in aqueous media. The discussion connects synthesis and structural regulation with reaction mechanisms, activity, selectivity, and stability. Particular attention is given to in situ and operando studies of Cu-based bimetallic systems for CO_2_ and nitrate reduction, complemented by ex situ characterization and theoretical analysis, to examine how Cu–M interfaces evolve under reaction conditions and influence catalytic performance.

## 2. Electrochemical Reduction of NO_x_

Methods for mitigating the impact of gaseous NO_x_ emissions include: (i) selective catalytic reduction (SCR), which is widely implemented in stationary applications [17]; (ii) selective noncatalytic reduction (SNCR) [18], used in specific contexts; and (iii) electrochemical reduction of NO_x_ (NO_x_RR), a technology currently under investigation [19]. Among these approaches, NO_x_RR offers several advantages: it can be conducted at ambient pressure and room temperature, is amenable to scale-up for industrial applications, and requires low energy input. Among several catalysts, Cu-based catalysts display exceptional performance because of the resemblance between the energy level of their d-band and the LUMO π* of NO_3_^−^ [20], tunable valence states, strong nitrate adsorption, and low cost, making them highly favorable candidates for electrocatalytic nitrate reduction reaction [21].

Noble metals such as Ir, Pt, Rh, Ru, Pd, Ag, and Au, as well as transition metals such as Cu, Fe, Co, and Ni, have demonstrated effectiveness for NO_x_ RR; however, their scarcity and high cost limit their feasibility for large-scale applications. Copper-based materials are promising alternatives because they exhibit low hydrogen evolution reaction (HER) activity and high electrocatalytic kinetics during the electrochemical reduction of nitrate (NO_3_^−^) species [22,23]. Consequently, copper-based nanomaterials are considered a promising, innovative, sustainable, and environmentally friendly alternative for NO_x_ elimination and sustainable ammonia (NH_3_) production [24]. These materials include Cu-single atom [25], Cu-monometallic [26], and Cu oxides [20,22,27,28,29,30]. Additional copper-based materials, including Cu-based alloys such as CuNi [31], CuPd [32,33], CuBi [34], CuCo [35], and CuRu [36], have been employed. Among these bimetallic alloys, ammonia synthesis performance is higher when a non-precious metal (Ni, Co, etc.) is introduced as a second active site with Cu, avoiding the use of precious metals such as Pd, Ag, Ru, and Rh. Finally, Cu nanoparticles supported on materials such as CeO_2_, fluorine-doped tin oxide (FTO) [37], and TiO_2_ [38], have also been investigated. Table 1 shows examples of Cu-based materials used as electrocatalysts for the NO_x_ electroreduction reaction from the literature.

### 2.1. NO_x_ Reduction Mechanism

Electrochemical reduction of NO_x_ is a complex process involving multiple electron and proton transfer reactions, as well as intermediate steps that lead to stable end products and residual nitrogen compounds such as NO_2_, N_2_, N_2_O, and N_2_H_4_. These residual compounds can decrease overall reaction efficiency and contribute to environmental pollution. A significant challenge in NO_x_RR is the relatively slow reaction kinetics, which may result from the low solubility of NO in aqueous media and the concurrent, competitive hydrogen evolution reaction (HER) [20,47]. Developing high-performance, durable catalysts for NO_x_RR that can effectively adsorb and convert a range of intermediates remains a significant challenge [34,48].

Cu-based materials show high electrocatalytic activity for the conversion of NO_3_^−^ to ammonia (NH_3_) compared with many other metal electrocatalysts because of several advantages, including high catalytic activity, relatively low operating potentials, low cost, tunable valence states, and structural versatility. Moreover, the energy level of the *d-*orbital of copper is close to the LUMO π* molecular orbital of nitrate, simplifying the electron transfer to the adsorbed nitrate and making Cu-based materials highly active. The initial step in nitrate electroreduction is kinetically controlled and involves the reduction of nitrate to nitrite (NO_2_^−^), largely due to the significant nitrate adsorption capacity of Cu. This step determines the overall reaction rate. Subsequent steps involve multiple intermediates, which complicates the elucidation of the reaction mechanism. The electrochemical conversion of nitrate to ammonia is inherently complex, as it requires the transfer of nine protons and eight electrons and may produce several by-products, including nitrite ions, nitrogen (N_2_), and hydrazine (NH_2_NH_2_). Wei et al. reported that the incorporation of foreign metal atoms into the copper crystal structure is influenced by both electrolyte pH and specific electrochemical reactions. In alkaline electrolytes, this process leads to the formation of nitrite (NO_2_^−^) species and hydroxylamine (NH_2_OH) [49].

According to the Frost–Ebsworth diagram, N_2_ and ammonia are among the most thermodynamically stable products; thus, both species could be obtained as final products according to reactions (1) and (2) [50]

2NO_3_^−^ + 12H^+^ + 10e^−^ ⟶ N_2_ + 6H_2_O (1)

NO_3_^−^ + 9H^+^ + 8e^−^ ⟶ NH_3_ + 3H_2_O (2)

The optimal electrocatalyst for NO_x_ reduction should exhibit the following properties: thermodynamic stability; efficient NO adsorption and activation, avoiding the hydrogen evolution reaction (or promoting proton-adsorption); high NO-reduction activity at a low limiting potential for NH_3_ formation; and high NH_3_ selectivity with effective inhibition of by-product formation [51].

Electrochemical impedance spectroscopy (EIS) is commonly employed to analyze the electrochemical reduction of NO_x_. For copper-based catalysts, existing studies have reported charge-transfer resistance using Nyquist plots. However, further investigation of the equivalent circuits that inform Nyquist plot interpretation is warranted, as such studies could provide deeper insight into electron-transfer processes during nitrate reduction on copper surfaces. The identification of liquid and gaseous products depends on the analytical technique used. Differential electrochemical mass spectrometry (DEMS), when coupled with cyclic-voltammetry polarization-profiles, enables real-time monitoring of generated products. In recent work, a PdCu/C electrocatalyst was used for the NO_x_ reduction reaction and coupled with online DEMS. This approach showed that the main species generated during the reduction reaction included N_2_, N^+^, NH_3_^−^, and N_2_H_4_, with hydrogen evolution observed at more negative potentials, which helped to propose a suitable reaction mechanism [33]. Finally, computational tools can be used to accurately simulate the reaction interface for NO_x_ electroreduction.

### 2.2. Key Factors in Designing Cu-Based Catalysts for NO_x_ Electroreduction

#### 2.2.1. Synthesis Method for NO_x_ Catalysts

Understanding the structure–activity relationship is fundamental to optimizing electrochemical performance. The method used to prepare catalytic materials determines their structural, morphological, crystalline, and compositional properties. For the preparation of Cu-based materials for NO_x_RR, solution-based and wet chemical synthesis methods are widely used because they favor the formation of Cu particles in three different oxidation states [52]. In addition, they can promote the formation of Cu^+^/Cu^0^ pairs, which are key to the kinetics of NO_3_^−^ to NO_2_^−^ conversion [39], and the exposure of specific crystalline facets that enhance catalytic activity [26,53]. Impregnation and chemical reduction methods are effective for the synthesis of Cu-based particles supported on carbonaceous materials [41] and MXenes [54,55], improving the dispersion of the active phase, catalytic activity, selectivity, and electron transfer. Electrodeposition is also a well-recognized method for depositing Cu-based nanoparticles, generally by employing Cu-containing electrodes or precursors [56,57].

The hydrothermal method offers several advantages, including low reaction temperatures, precise compositional control, high purity, and uniformity in particle size and shape [58]. The composition of hydrothermally processed catalysts allows the preparation of alloys [40] and doped Cu-based materials [59]. Post-synthesis thermal treatment is often employed to modulate Cu oxides features.

Hao et al. [40] synthesized bimetallic copper-iron nanosheets in alkaline media (KOH) using hydrothermal processing, followed by calcination at 300 to 500 °C in a reducing atmosphere of 10% hydrogen in argon. During calcination, the hydrothermally treated 25 nm thick CuFeO_2_ nanosheets were transformed into a mixture of copper and iron nanoparticles (5–10 nm) distributed within the nanosheet structure. Materials calcined at 450 °C exhibited the highest number of exposed active sites and achieved the greatest ammonia production rates from nitrate and nitrite electrolytes. Calcination under an Ar atmosphere was employed by Shuyi Shen et al. [59] to transform hydrothermally synthesized CuCo(OH)_x_ into CuCo_3_O_x_ catalyst. The reaction occurred at 120 °C for 6 h, employing metallic nitrates and urea as precursors. The catalyst calcined at 650 °C exhibited strong electrochemical performance in NH_3_ production.

Sol–gel synthesis of Cu oxides is typically conducted in aqueous or alcoholic solutions using hydrolysis and gelling agents [39,60] and synthesized CuO and Cu-phosphate catalysts by a sol–gel method using phosphate salts as precursors. After calcination at 600 and 700 °C, the materials were evaluated for electrochemical NO_3_^−^ reduction, with CuO showing the best NH_3_ yield among the materials tested. Bai et al. [22] reported the synthesis of Cu_2_O nanoparticles by partial reduction of Cu^2+^ ions. They achieved surfactant-free partial reduction of CuSO_4_ to Cu_2_O using L-ascorbic acid in an alkaline NaOH solution, resulting in 20–40 nm Cu_2_O nanocubes after 15 min at room temperature. The Cu^+^ species drives the nitrate-to-nitrite reduction.

#### 2.2.2. Effect of Valence State of Cu Species for NO_x_ Catalysts

Copper-based catalysts are widely considered ideal candidates for the nitrate reduction reaction due to the analogous energy level properties of Cu d-orbitals and the lowest unoccupied molecular π* orbitals of nitrate. Thus, Cu-based catalysts and Cu species reconfigure to act as active sites for the nitrate reduction process.

Shanna An et al. used CuO and Cu-based phosphates as precatalyst for the electrocatalytic nitrate reduction reaction. The electrochemical reconstruction of these precatalysts generates active Cu species with tunable valence states for NO_3_^−^ conversion where CuO exhibits NH_3_ yield of 8.16 mg h^−1^ mgcat-1 with a Faradaic efficiency of 95.72% at −0.95 V (vs. RHE) compared to Cu_2_P_2_O_7_ (7.33 mg h^−1^ mgcat^−1^ and Faradaic efficiency of 94.88%) and Cu_3_(PO_4_)_2_ (6.53 mg h^−1^ mgcat^−1^, Faradaic efficiency of 92.04%). In situ Raman and XPS reveal that electrochemical reconstruction of copper species generates distinct active sites such as Cu^1+^/Cu^0^ (CuO-derived) and Cu^2+^/Cu^0^ pairs (CuPO-derived). Additionally, operando differential electrochemical mass spectrometry and in situ Fourier transform infrared spectroscopy revealed that the Cu^1+^/Cu^0^ active pairs have fast catalytic kinetics for the conversion of *NO_3_^−^ to *NO_2_^−^ [39].

Ting Sun et al. reported that the morphologies and valence states of Cu species affected their NO reduction performance. Cu nanoparticles on nanofibers adopted an octahedral structure, and CuO nanosheets formed ball clusters. The sheet-like CuO within these ball clusters promoted the concentration of the electric field at sharp tips, favoring efficient capture and electroreduction of NO molecules near the electrode. CuO-nanofibers exhibited a NH_3_ yield of 0.5 mg cm^−2^ h^−1^ at −0.6 V (vs. RHE) with a Faradaic efficiency of 29.68%, higher than that of Cu-nanofibers (0.17 mg cm^−2^ h^−1^, and Faradaic efficiency of 16.18%) [30].

#### 2.2.3. Alloying and Doping Effects in Cu-Based NO_x_ Catalysts

Cu alloys exhibit a regulated electronic structure after introducing another metal into the Cu crystal structure. The ability of Cu to adsorb *H is weak. Thus, incorporating other metals tunes the affinity of different intermediates, reducing the reaction barrier and onset potential and enhancing activity toward the nitrate electroreduction reaction. Transition metals such as Cu, Fe, Co, and Ni have favorable electrocatalytic activity and cost-effectiveness.

Recently, Junbeom Maeng et al. reported a Cu-Fe dual atom catalyst anchored on nitrogen-doped carbon for the electrochemical nitrate reduction reaction. The authors found that atomically dispersed Cu and Fe ensemble sites with strong N coordination promote electronic interaction and structural stability, showing a faradaic efficiency of 94.3% and an NH_3_ production rate of 6 mg·cm^−2^·h^−1^ at 0.53 V (vs. RHE). Finally, the mechanism indicated that Cu sites initiate NO_3_^−^ activation, while Fe sites facilitate NO_2_^−^ hydrogenation [61].

Lately, Yuxiao Liu et al. designed a copper single atom (Cu-N_3_) coupled with an adjacent iron cluster (Fe_3_-N_8_) decorated on nitrogen-doped carbon, and in situ characterizations revealed that the catalyst structure assists electron redistribution within trinuclear Fe cores while preserving Fe-N_4_ activity, and this creates a special charge polarization gradient through electron-deficient Cu sites and electron-rich Fe clusters that promotes abundant H* generation at Fe sites and NO_3_^−^ adsorption and activation at Cu sites. This synergistic effect in the bimetallic catalyst produced highly efficient electrochemical nitrate reduction to NH_3_, with a Faraday efficiency of 97.1% and an NH_3_ yield of 18.83 mg·h^−1^·mgcat^−1^ at −0.65 V (vs. RHE) [62].

In addition, Shuyi Shen et al. reported a CuCo bimetallic catalyst synthesized via the hydrothermal method and followed by controlled annealing (CuCo_3_O_x_-650) that exhibits a Faradaic efficiency of 90.8% and NH_3_ generation of 312.1 µmol h^−1^ cm^−2^ at −0.5 V, which was analyzed by density-functional-theory; this technique indicated that Co atoms are the principal channels for charge transfer during NO adsorption on CoCuO_2_. Incorporating Cu induces additional electron redistribution, shifting the *d*-band center. The authors indicated that the adsorption energy of *H on CuCo_3_O_x_-650 was 1/7 that for NO, which suppresses the competing HER by minimizing *H accumulation. This suggests that the synergistic effect of Cu and Co improves activity and selectivity for NO-to-NH_3_ conversion [59].

Yuhang Wang et al. prepared CuNi catalysts with Cu:Ni ratios of 80:20, 50:50, and 30:70. Their findings showed that the Cu_50_Ni_50_ alloy catalyst (Faradaic efficiency 99 ± 1% for NH_3_ at −0.15 V vs. RHE) substantially improved nitrate electroreduction. The introduced Ni atoms shifted the potential-dependent step from NO_3_^−^ adsorption to *NH_2_ hydrogenation due to the enhanced adsorption energy of NO_3_^−^ on the CuNi surface, consequently decreasing the overpotential. Additionally, Ni alloying modifies the Cu *d*-band center and modulates the adsorption energies of intermediates such as *NO_3_^−^, *NO_2_, and *NH_2_ in alkaline conditions [31].

In addition, the insertion of noble metals such as Pt, Pd, Au, Ru, and Rh has been investigated to improve the behavior of Cu-based catalysts towards NO_x_ electroreduction. For instance, Weimian Zhang et al. prepared atomically dispersed isolated Pt atoms on Cu nanowires to obtain a Pt_1_-Cu single atom alloy that exhibits a Faradaic efficiency of 92.4% and an NH_3_ yield rate of 16.4 mg cm^−2^ h^−1^. They verify the in situ generation of reactive *H species on Pt sites. The atomically dispersed Pt facilitates nitrate adsorption and hydrogenation and, at the same time, suppresses nitrite accumulation [63].

Hongbo Zhu et al. reported Pd-nanoparticle catalyst systems with different sizes (0.28, 2.88, and 9.2 nm) supported on Cu foam for the nitrate electroreduction reaction. They found that the H* coverage can be adjusted by changing Pd nanoparticle sizes in the Pd@Cu system, and Pd nanoparticles having an average size of 2.88 nm can promote NO_3_^−^ hydrogenation to NH_3_ via well-modulated coverage of adsorbed H* species, showing the best activity for the nitrate reduction reaction with a Faradaic efficiency of 97% at −0.8 V (vs. RHE) and a NH_3_ yield 21 mg h^−1^ cm^−2^ [64].

Additionally, Shuchun Sun et al. explored the behavior of a bimetallic alloy Au_3_Cu catalyst supported on carbon for the NO_3_^−^ to-NH_3_ conversion. The catalyst exhibits high activity attributed to the synergistic effect between Au and Cu sites, finding that Cu shows selectivity in the reduction of NO_3_^−^ to *NO, and Au evidence performance in the next step of reduction, *NO to NH_3_. The authors also confirm that d-d orbital hybridization adjusted the d-band center of the Au_3_Cu alloy. Finally, they corroborate a NH_3_ generation rate of 1719.3 µg h^−1^ cm^−2^ and a Faraday efficiency of 95.1 at −0.5 V (vs. RHE) [65].

Zi-Xin Ge et al. synthesized polyallylamine-functionalized frame-like concave RhCu bimetallic nanocubes exhibiting improved electroactivity and selectivity for the nitrate electroreduction reaction in acidic solution, with a Faradaic efficiency of 93.7% and an NH_3_ yield rate of 2.40 mg h^−1^ mg_cat_^−1^. Experimental and theoretical calculations showed that the Rh electronic structure/adsorption capacity was modified by Cu and polyallylamine, and polyallylamine enhanced the interfacial mass transfer process through electrostatic interactions, which are responsible for the intrinsic reaction activity and selectivity of polyallylamine-RhCu nanocubes [66]. In contrast, Wensheng Gao et al. synthesized Cu-Ru alloys with varying proportions supported on reduced graphene oxide (rGO) to investigate the synergistic effect of these elements through their active sites in NO_3_^−^ reduction. Incorporating Ru into Cu shifts the d-band center of the alloy and modulates the adsorption energy of NO_3_^−^ and NO_2_^−^, promoting the reduction of NO_3_^−^ to NH_3_. Ru_1_Cu_10_/rGO catalyst showed an ammonia formation rate of 0.38 mmol cm^−2^ h^−1^ and a Faradaic efficiency of 98% at −0.05 V (vs. RHE) [67].

As previously mentioned, the electrocatalytic nitrate reduction involves multiple electron and proton transfers and intermediates such as *NO_3_, *NO_2_, *NO, etc. This makes the selective conversion to NH_3_ a challenge because of the inability of most of the catalysts to adsorb and activate the oxygen-containing intermediates. Thus, NO_3_^−^ preferentially adsorbs onto the active site through oxygen atoms with the free lone pair electrons, whereas *NO_2_ and *NO adsorb via nitrogen or oxygen atoms; therefore, the adsorption configuration establishes the reaction pathway and final product selectivity. Consequently, the design of high-performance nitrate electroreduction catalysts also focuses on active sites that can accomplish the adsorption of diverse intermediates, using metal doping strategy to influence adsorption energies and drive reaction pathways. Element doping can modify the catalyst’s crystal and electronic structures, affecting local charge distribution and changing the surface electronic structure of the Cu-based catalyst. This change affects the electron affinity of the catalyst surface, adjusting the energy levels of the active sites. Hexing Lin et al. reported that the incorporation of Bi into the CuCo_2_O_4_ catalyst lowers the reaction barrier for the hydrogenation step from *NO_2_ to *NO_2_H, promoting the mass transfer in the liberation of *NH_3_ and the reactivation of the active sites. Thus, Bi_1_CuCo_2_O_4_ catalyst shows a Faradaic efficiency of 95.53% and a NH_3_ yield of 448.74 µmol h^−1^ cm^−2^ at −0.8 V vs. RHE [68]. Systems such as Zn-doped Cu exhibit a Faradaic efficiency of NH_3_ of 98.4% and a yield rate of 5.8 mol g^−1^ h^−1^ [69], and recently, Bi-doped Cu with a Faradaic efficiency of 93.1% at −0.8 V vs. RHE has been shown to favor the conversion of NO_3_^−^ to NH_3_ [34].

#### 2.2.4. Morphological and Crystallographic Facets Effects in Cu-Based NO_x_ Catalysts 

Novel strategies have been developed to improve the electrocatalytic performance of Cu-based electrocatalysts. Between them, crystal facet and morphology engineering play a key role in determining electrocatalytic performance of nitrate reduction.

Jui-Yao Chang et al. synthesized a Cu/Cu_2_O heterostructure electrocatalytic membrane for electrospun-derived activated carbon fibers via the electrodeposition method. The authors followed the crystal facet ratios of both Cu and Cu_2_O by electrodeposition time which influenced electrocatalytic performance. The authors showed that a lower Cu_2_O (200)/Cu_2_O (111) facet ratio suppressed the HER and promoted NO_3_^−^ to NO_2_^−^ conversion due to the high concentration of oxygen vacancies and hydroxyl groups at the surface. Moreover, a higher Cu (200)/Cu (111) facet ratio reduced the onset potential for both NO_3_^−^ to NO_2_^−^ reduction, enhancing the overall nitrate reduction efficiency and selectivity. Thus, the highest ammonia selectivity of 91.54% and an ammonia yield rate of 3.96 µmol h^−1^ mg_cat_^−1^ were exhibited with the electrocatalytic membrane with an elevated Cu (200)/Cu (111) facet ratio [70].

Jiaqui Shao et al. reported the synthesis of CuO nanosheets by oxidation of electrodeposited Cu nanoparticles. CuO nanosheets with high specific area and density defects conformed snowflakes structures, where nanosheets stack at the center. No H_2_ generation was detected; however, small quantities of N_2_ and N_2_O appear. The catalyst exhibited a maximum ammonia Faradaic efficiency of 92.1% at 0.02 V (vs. RHE), suppressing the formation of N_2_ and N_2_O. At negative potentials (−0.26 V vs. RHE) current density reached 1.1 A/cm^2^ with ammonia Faradaic efficiency of 89.5%. The catalyst showed stability during 50 h at 0.19 V (vs. RHE) with a current density up to 400 mA cm^−2^ and a Faradaic efficiency of 80% [56].

On the other hand, preferential adsorption of NO_3_^−^ on the catalyst surface also plays a critical role in enhancing the NH_3_ selectivity of nitrate electroreduction. Haiyan Ma et al. showed that copper oxide catalyst (Cu-B_2_) has dendritic morphology exhibiting stability after long-term (12 h) nitrate electroreduction, reaching a current density of 192.3 mA cm^−2^ at −0.6 V (vs. RHE) with Faradaic efficiency of 94% at −0.6 V (vs. RHE) and 16.9 mg h^−1^ cm^−2^. In situ attenuated total reflectance surface-enhanced infrared spectroscopy studies have shown that the Cu-B_2_ catalyst displays a potential-independent adsorption capability of the NO_3_^−^ reactant, which promotes conversion of the NO_2_^−^ intermediate into NH_3_ [29].

Dongdong Wang et al. recently reported Cu-Co dual sites in cobalt oxide nanoboxes for the electrocatalytic reduction of NO to NH_3_. Thus, Cu-Co_3_O_4_ nanoboxes and Cu-Co_3_O_4_/CuO nanoboxes with Cu modification show a Faraday efficiency of 93% and an ammonia yield of 59.10 µg h^−1^ mg_cat_^−1^ at −0.4 V (vs. RHE). The physical characterization and density functional theory results showed that NO adsorption energies on the surfaces of Co_3_O_4_ (311), Cu- Co_3_O_4_ (311) and Cu- Co_3_O_4_ (311)/CuO (002) are −1.81, −1.95 and −2.27 eV, respectively, pointing out an enhancement in NO adsorption ability after formation of the Cu-Co dual sites [71].

Yuankang Zha et al. showed that Au-doped Cu nanowires grown on a copper foam showed ammonia production rates of 5336 µmol h^−1^ cm^−2^ and Faradaic efficiency of 84.4% at −1.05 V (vs. RHE), and pH of 5.6. The presence of oxygen vacancies generated during thermal treatment induces the N-O bonding weaking. Also, Au atoms promote electron transfer with Cu^0^, Cu^+^ and Cu^2+^ during NO_3_^−^ reduction, enhancing charge density and inhibiting the production of H_2._ XPS and in situ infrared spectroscopy results pointed out that the electron transfer at the Au-Cu interface and oxygen vacancies synergistically lowered the reduction reaction barrier and inhibited hydrogen generation, improving conversion and selectivity for nitrate electroreduction [57].

Cu nanosheets in situ derived from CuO nanosheet catalysts showed stability for 700 h, with a Faradaic efficiency of 88% at −0.59 V (vs. RHE) and 365 mA cm^−2^. The performance is attributed to the interaction of Cu (100) and Cu (111) facets. The NO_2_^−^ generated on the Cu (100) facets is then hydrogenated on the Cu (111) facets. Thus, both catalysts favor the hydrogenation of *NO to *NOH for NH_3_ production [72].

Lei Xiao et al. synthesized Cu nanosheets with exposed (111) surfaces and reported a NO-to-NH_3_ yield of 371.89 µmol cm^−2^ h^−1^ at −0.59 V (vs. RHE) in a flow cell, with a Faradaic efficiency of 93.19 ± 1.73% by selectively exposing Cu metal crystal surfaces. The key intermediate NO^−^ was observed during the adsorption process of NO on Cu (111) surfaces using Operando attenuated total reflection infrared spectroscopy, and simulation results elucidated the overall reaction pathway as *NO ⟶ *NOH ⟶ *N ⟶ *NH ⟶ *NH_2_⟶ *NH_3_ over a Cu (111) catalyst [26].

Finally, Yu-Jen Shih et al. explored Cu nanoparticles synthesized via electroplating in the presence of surfactants and supported on stainless steel mesh electrodes. The nature and concentration of surfactants affected the crystal facet of Cu particles. The ratio of Cu(200)/Cu(111) controlled the selectivity of N_2_, NO_2_^−^ and NH_4_^+^. Pd incorporated in Pd-Cu on stainless steel enhanced nitrogen selectivity due to hydrogenation [73].

#### 2.2.5. Overview of Contributing Factors Affecting NO_x_ Catalysis

The electrocatalytic reduction of NO_x_ is governed by the combined effects of catalyst composition, surface structure, reaction conditions, and catalyst evolution under operating conditions. Although oxidation states, Cu alloying, morphology, and exposed Cu facets influence activity, these factors alone do not explain differences in activity, selectivity, and stability. However, the catalytic behavior of Cu-based materials is sensitive to the interaction between the Cu surface and its local environment. A key factor is the ability of Cu to provide multiple types of adsorption sites depending on its oxidation state, the incorporation of other elements, morphology, and the exposed surface facet. Selectivity is determined by competition among parallel processes such as HER, reduction of nitrogen-containing intermediates, and reduction of dissolved oxygen, among others. The contribution of these pathways depends on species’ surface coverage and local availability. The stability of Cu-based catalysts is also an important factor because during electrolysis, Cu nanoparticles, oxides, and Cu-based alloys may undergo restructuring, agglomeration, changes in oxidation state, etc. These processes can change the electrochemically active surface area and expose new active sites. Finally, all these differences can contribute to the variability of Cu-based catalysts with relatively high Faradaic efficiency. However, careful selection of factors such as synthesis method, catalyst loading, NO_x_ concentration, alloying elements, catalyst morphology, etc., is required. Thus, the performance of Cu-based catalysts in multivariable processes should be followed using appropriate in situ and ex situ techniques to fully understand this important NO_x_ reduction alternative.

## 3. Electrochemical Reduction of CO_x_

The atmospheric concentration of CO_2_ continues to rise, intensifying the greenhouse effect and contributing to global warming. One proposed solution is to capture CO_2_ emissions before they enter the atmosphere, then reduce them electrochemically in aqueous media. This process enables the production of value-added products such as carbon monoxide (CO), formic acid (HCOOH), methane (CH_4_), ethylene (C_2_H_4_), ethanol (C_2_H_5_OH), ethane (C_2_H_6_), and formate (HCOO^−^). These compounds serve as feedstocks for industrial chemical synthesis, energy generation, or as fuels.

CO_x_RR can be achieved using various polycrystalline metal electrodes. Previous studies indicate that Pb, Hg, Tl, In, Sn, Cd, and Bi mainly yield formate species (HCOO^−^) [74]. In contrast, Au, Ag, Zn, Pd, and Ga predominantly produce carbon monoxide (CO), whereas Ni, Fe, Pt, and Ti are inefficient for CO_2_ reduction and instead facilitate the reduction of H_2_O to H_2_ [75]. Also, Pd catalysts can yield formate [76].

Copper electrodes, however, generate hydrocarbons, aldehydes, and alcohols [77]. Consequently, a key challenge is developing electrocatalysts that are selective, active, low-cost, and stable. Table 2 summarizes Cu-based catalysts used for CO_x_RR, highlighting the versatility of catalyst design approaches and the electrochemical conditions in CO_x_RR.

### 3.1. CO_x_ Electrochemical Reduction Mechanism

Electrochemical reduction of CO_2_ (CO_x_RR) can follow two well-known pathways: electron–electron–proton–proton (EECC) and electron–proton–electron–proton (ECEC) [77,86]. In both cases, the general reaction is written in Equation (3); the products include C1, C2, and C2+ species.

CO_2_ + *n*H^+^ + *n*e^−^ ⟶ Products + H_2_O (3)

Copper is the only metal capable of reducing CO_2_ to both C1 and C2 products, such as CO, methane (CH_4_), ethane (C_2_H_4_), formate (HCOO-), acetate, and alcohols in aqueous media. This unique behavior is attributed to copper’s ability to suppress the hydrogen evolution reaction, thereby enhancing CO intermediate production, consistent with the findings of Zeng et al. [87]. Furthermore, the distribution of products from the electrochemical reduction of CO_2_ in aqueous media depends on multiple parameters, including the catalyst, surface engineering, coordination control, catalyst/electrolyte interface environment, pH, morphology, electrolyte composition, particle size, crystallite orientation in the copper matrix, surface roughness, electrode potential, overall composition, CO_2_ diffusion mechanism, and particle size [83,87,88,89]. The selection of electrolyte plays a critical role in CO_2_ reduction, as the local reaction environment—including the composition and concentration of anions and cations—modifies electrostatic interactions, buffer capacity, pH, and the availability of proton donors. Studies indicate that cation size strongly determines the selectivity of CO_2_ and CO electroreduction on polycrystalline copper. Larger cations enhance selectivity for C2 species formation while decreasing H_2_ production [90]. CO_2_ reduction catalysis is commonly studied in KHCO_3_ electrolytes because the CO_2_-bicarbonate-water equilibrium maintains a neutral pH. Hori et al. reported that electrolytes such as KCl, KClO_4_, K_2_SO_4_, and KHCO_3_ favor the formation of hydrocarbons and alcohols, whereas K_2_HPO_4_ solutions promote H_2_ production [91,92]. Liquid products generated during the electrochemical CO_2_ reduction are typically analyzed using high-performance liquid chromatography (HPLC), ion chromatography (IC), nuclear magnetic resonance (NMR), and chemical oxygen demand (COD). In contrast, gaseous products are commonly analyzed by gas chromatography–mass spectrometry (GC-MS) [88].

However, there is a lack of studies that monitor the production of gaseous products in real time using techniques such as in situ differential electrochemical mass spectrometry (DEMS), which could enable direct observation of gaseous species at the site of electroreduction (in situ). Such methods could help address challenges in identifying species generated during the electrochemical reduction of carbon oxides. Electrochemical CO_2_ reduction on copper catalysts is promising; however, several challenges must be addressed before scalable technology can be realized. These include rational catalyst design, a fundamental understanding of CO_2_ reduction mechanisms, the development and implementation of in situ and ex situ characterization techniques [93], and the validation of theoretical predictions for catalyst design [94,95].

Determining the reduction mechanism is a difficult task, and many studies have reported theoretical calculations. Tao Cheng et al. [96] carried out QM calculations of CO_2_RR on Cu(100) in water, producing C1 species, CO, and formate (HCOO^−^). First, CO_2_ is physisorbed and then chemisorbed (*CO_2_^δ−1^) as seen in Equation (4) with an energy barrier of ΔG^++^ of 0.43 eV. After, *CO_2_^δ−1^ is protonated forming COOH* (ΔG = 0.37 eV, Equation (5)) and then dissociated to *CO (ΔG = 0.30 eV, Equation (6)). On the other hand, HCOO^−^ is produced by direct reaction of physisorbed CO_2_ with adsorbed H* (ΔG = 0.8 eV) as observed in Equation (7). Cu(100) promotes selectivity to CO production when the determining step is the first electron-transfer.

(4) $$\mathrm{CO}\;\mathrm{formation}:\;{\mathrm{CO}}_{2}\left(\mathrm{aq}\right)\overset{\delta e^{-}}{\to }{\mathrm{CO}}_{2}^{\delta -}$$

(5) $${\mathrm{CO}}_{2}^{\delta -}\overset{H_{2}O+e^{-}}{\to }*\mathrm{COOH}$$

(6) $$*\mathrm{COOH}\begin{array}{c} e^{-}\\ \to \\ {\mathrm{OH}}^{-} \end{array}*\mathrm{CO}$$

(7) $$\mathrm{Formate}\;\mathrm{formation}:\;{\mathrm{CO}}_{2}\left(\mathrm{aq}\right)\overset{\;H^{.}+{\;e}^{-}\;}{\to }{\mathrm{HCOO}}^{-}$$

C-C coupling is key to producing C2 and C2+ chemicals. Zhang and Wang [97] simulated a multi-pathway C-C coupling under different voltage and pH conditions using DFT. They proposed a microkinetic model that treats CO as the starting species and found that applied voltage and local pH modify intermediate coverage and stability, guiding the reaction along different routes. As shown in Figure 3, 6 C1 condition (pH = 13) intermediates and 21 C-C coupling combinations were found. Under alkaline and low applied potentials, oxygenated intermediates such as *COH and *CHOH are prominent. In contrast, at high applied voltage, the hydrogenation increases, favoring the coupling of hydrocarbon intermediates (CH and *CH_2_) that conduct ethylene formation.

### 3.2. Key Factors in Designing Cu-Based Catalysts for CO_x_ Electroreduction

Different strategies are employed to design Cu-based catalysts to improve activity, selectivity, and stability, and to guide the CO_x_RR mechanism. These strategies help stabilize intermediates, increase CO coverage, promote C-C coupling, and control the distribution of C1 and C2 products.

#### 3.2.1. Synthesis Method for CO_x_ Catalysts

The bibliography reports the synthesis of metal-based materials used in CO_x_RR by physical methods and, especially, by chemical methods such as chemical reduction, electrochemical deposition, precipitation, ligand displacement and hydrothermal methods, among others. All of them offer specific features of Cu-based catalysts for CO_x_RR.

Chemical reduction and electrochemical deposition stand out among wet-chemical methods for their versatility and precise control of morphology, defect density, and the oxidation state of Cu-based materials for CO_x_RR [98]. Chemical reduction implies the reduction of metal ions in suitable polar or nonpolar solvents. Typically, a stabilizing agent is introduced to prevent agglomeration or cap the metallic nanoparticles. Key experimental parameters, including the precursor-to-reductant-to-stabilizer ratio, pH, homogenization time, and temperature, significantly influence the characteristics of the resulting nanoparticles. Granular copper nanoparticles (50–60 nm) with a Cu (111) orientation were evaluated for CO_2_ electrochemical reduction, producing ethylene, formic acid, ethanol, and formaldehyde. Cu nanoparticles show selectivity to ethylene and aqueous products such as ethanol, formic acid, and formaldehyde (−1.3 V vs. RHE) [84]. Incorporating ionic liquids with copper nanoparticles shifts selectivity toward methanol. Luc et al. [99] reported the synthesis of triangular two-dimensional Cu nanosheets with selectively exposed (111) planes. Cu reduction was modulated by employing hexadecyltrimethylammonium bromide (CTAB) and hexamethylenetetramine (HMTA). This approach resulted in a trigonal planar geometry and isotropic growth of 5 nm thick Cu nanosheets. These materials selectively reduced CO to acetate (FE of 48%, 131 mA cm^−2^ in 2 M KOH electrolyte), inhibiting the formation of ethylene and ethanol by reducing the exposed (100) and (110) surfaces. Bimetallic nanoparticles were synthesized by introducing both metal salts into the reaction system. Xiong et al. [100] reported the production of Pd@Cu nanostructured coral-like particles using palladium chloride and copper nitrate in an ethanol-deionized water solution, with reduction proceeding for 2.5 h at 60 °C. Additionally, Dahal et al. [101] reported morphology-controlled Cu_2_O octahedrons-nanoparticles synthesis by gradually dissolving ice cubes of Cu(NO_3_)_2_ solution at varying concentrations into NaBH_4_ in ethanol. The concentration and gradual release of Cu^2+^ ions at pH 4.4 determined the size and morphology of the resulting powders. The FEs for CO, CH_4_, and C_2_H_6_ (−0.3 V vs. RHE) were 11.90, 76.61, and 1.87, respectively. Modulation of morphology is frequently achieved by employing surfactants, as proposed by Bekey et al. [102] in the electrodeposition of Cu nanoparticles. Nubby structures are obtained if no sodium dodecyl sulfate is employed, while the addition of the surfactant produces dendritic particles. Also, H and flow cells were employed to determine the FE of Cu^0^/Cu^+^ interfaces grown by electrodeposition on carbon paper at different times [103]. The catalyst deposited for 5 min reaches an FE of 61.2% (−0.88 V vs. RHE) for C2+ and 62.0% (−1.265 V vs. RHE) in a flow cell.

Other wet-chemical syntheses can modulate morphology, facets, defect density, etc., to modify selectivity. Suliman et al. examined the performance of Cu-based nanomaterials prepared by co-precipitation in the electroreduction of CO_2_ [104]. They synthesized Cu and AgCu complexes with melamine by mixing ion precursors dissolved in ethanol, followed by purging with N_2_ for 14 h at 100 °C. The resulting products exhibited semi-cubic morphologies approximately 30–50 nm in size. The Cu–melamine complex showed selectivity for C1 products. The optimal AgCu–melamine complex contained 10 wt.% Ag. It favored C2 products with an FE of 70% at −1.0 V vs. RHE and a current density of −50 mA cm^−2^ [105]. First, solvothermal synthesis was conducted using CuCl_2_, dopamine hydrochloride, and ammonium dihydrogen phosphate in an ethanol–water solvent at 150 °C for 4 h. Subsequently, pyrolysis for 2 h in a nitrogen atmosphere at temperatures between 500 and 900 °C was performed. Dopants promoted the dispersion of copper atoms within the carbon matrix. Phosphorus doping suppresses the hydrogen evolution reaction and enhances ethylene and ethanol production. Cu^+^-Cu^0^ coexistence and Cu single atoms determine C-C coupling. Yan et al. [106] examined the selectivity of CuO nanosheets supported on graphite-phase carbon nitride (CuO/g-C_3_N_4_) for the electrochemical reduction of CO_2_ to C2 products. CuO nanosheets alone show lower activity and selectivity compared to the CuO/g-C_3_N_4_ composite. CuO was synthesized using Cu(NO_3_)_2_ and NaOH as precursors, with polyvinylpyrrolidone and sodium dodecyl sulfate as capping and stabilizing agents; the reaction was conducted at 170 °C for 24 h. FE reached 64.7% for C2 products and 37.0% for ethylene (−1.0 V vs. RHE).

#### 3.2.2. Effect of Valence State of Cu Species for CO_x_ Catalysts

The electronic properties of Cu make it an excellent catalyst because the coexistence of Cu^0^/Cu^+^/Cu^+2^ enables charge transfer and adsorption of specific intermediates in CO_x_RR. Li et al. [107] reported on a Cu-CuI composite catalyst where the abundant Cu^0^/Cu^+^ interfaces are responsible for high-rate CO_2_RR to C2+ products, mainly ethylene and ethanol. The composite was produced by simply mixing Cu nanoparticles and CuI powders. During CO2RR in a KOH electrolyte, the catalyst achieves a partial current density of 591 mA cm^−2^ (−1.0 V vs. RHE) and an FE of 71%. Stability was tested for 85 h at 550 mA cm^−2^. According to DFT calculations, the high-rate C2+ production on the composite is attributed to residual Cu^+^ and adsorbed I, both of which improve CO adsorption and C-C coupling. By performing quasi in situ XPS and ex situ AFM, Arán-Ais et al. [108] determined that continuous regeneration of Cu^+^ and defects on Cu (100) synergistically promotes C-C coupling. During pulsed CO_x_RR on a Cu(100) single-crystal electrode, an FE of 76% is reached at −1.0 V (vs. RHE) to ethylene, ethanol and n-propanol. DEMS confirmed the products and intermediates. Ethanol selectivity is linked to the coexistence of Cu^+^ and Cu^0^, while ethylene production is linked to the length of the Cu (100) terraces.

Chou et al. studied the role of the oxidation state of Cu on CO_2_RR [109]. In situ surface-enhanced infrared absorption spectroscopy (SEIRAS), soft X-ray absorption spectroscopy (XAS), and online gas chromatography were employed to determine the nature of adsorbed CO_2_ and the oxidation state of the catalysts. The Cu electrodes were prepared by physical vapor deposition, depositing a 36 nm thickness thin film on Au or Si substrates. The oxidation state of Cu in the electrodes was set by oxidation. The as-prepared Cu electrode (Si substrate) with Cu^0^ as the predominant species produces H_2_, CO and formic acid with FEs of 50, 5 and 10%, respectively (−1 V vs. RHE in 0.1 M KHCO_3_ electrolyte). If Cu^+^ predominates on the electrode surface, adsorbed CO mainly reduces to methane (FE of 30%) and formic acid (FE 8%) but mainly to H_2_ with an FE of 55%. In contrast, the Cu^0^+Cu^+^ mixture promotes C-C coupling to ethylene formation (FE 40%); methane is also produced (FE 35%).

#### 3.2.3. Alloying and Doping Effects in CO_x_ Catalysts

Bimetallic Cu alloys are currently employed for CO2RR in the production of CO [100,110]. Cu alloying with In and Sn improves CO_2_RR selectivity to CO at elevated temperatures (40–70 °C) [111]. Small quantities of In (1.5 at%) and Sn (4.2 at%) improve the FE of Cu alloy at 60 °C. FE of the InCu alloy reached >95% for CO at 1100 mA cm^−2,^ with reduced contribution from H_2_ (<4%) and C2+ (<12%). A higher FE is obtained from Sn-Cu catalyst, reaching 98% in a wide current density window (100–1100 mAcm^−2^) but after 80 h FE decreases to 95% at 500 mA cm^−2^. In situ ATR-SEIRAS analyses revealed that at 60 °C, the SPET (sequential proton–electron transfer) and hydrolysis were occurring according to (a) CO_2_ + E^−^ → *COO^−^ and (b) *COOH → *CO. Xu et al. reported on In doping where In acts as the principal active site in 30 nm In-coated CuO nanowires prepared by in situ electroreduction. A high FE of CO, 91% at a geometric current density of −69 mA cm^−2^, was attained [112]. Raman in situ indicates that small coating thickness promotes the adsorption of *CO, raising C2+; however, 30 nm In coating avoids the adsorption of *CO and the formation of *CO^2−^. Furthermore, Peerlings et al. [113] proved the coexistence of spatially separated CuO and ZnO in Cu_1−x_Zn_x_O catalysts grown on carbon paper. Carbon paper was placed in a Teflon-lined stainless steel autoclave containing Cu(NO_3_)_2_ and Zn(NO_3_)_2_ as precursors, and hexamethylenetetramine as a stabilizer. Electrodes composed of bimetallic oxides showed higher activity for CO production than those having individual oxides. In situ X-ray absorption spectroscopy (XAS) revealed that ZnO suppresses ethylene production on Cu while favoring CO production on ZnO.

Doping is a current strategy to control absorption, stabilize Cu species, and modify selectivity. Al doping of CuO is proposed by Tang et al. [114] to stabilize Cu^+^ during catalyst reconstruction in CO_x_RR. Cu^+^/Cu^0^ proportion is higher in AlCuO_x_ than in pure CuO_x_. Al shortens the Cu-O bond, stabilizing Cu^+^. This material shows good electrocatalytic performance for ethylene and ethanol production with an FE of 73.6% at −221 mA cm^−2^. Doping Cu-based catalysts with halogens enhances selectivity to C2+ products; their electronegativity and electronic nature can modulate the charge density and valence state of Cu-based catalysts [115,116]. Wenchao Ma et al. [115] determined that FE and yield to ethylene and ethanol decrease with halogen electronegativity doping in the order F-Cu > Cl-Cu > Br-Cu > I-Cu. DFT calculations indicate that adsorbed F^−^ on Cu_2_O, processed by a hydrothermal-calcination process, limits HER because it avoids the dissociation of adsorbed H_2_O molecules. Also, F- promotes the hydrogenation of CO to *CHO which dimerizes to ethylene. The catalyst achieves an FE of 40.6% (−1.8 vs. RHE) and maintains it for 13 h. The uncalcinated material reaches only 19.7% FE, while Cu foil attains only 3.7% [116].

#### 3.2.4. Morphological and Crystallographic Facet Effects in CO_x_ Catalysts

Morphology strongly affects facets, surface area, electrochemical active surface (ECSA), quantity of active sites, etc. [117,118]. These features determine the catalyst performance. Cu nanosheets with high defect density were successfully employed for ethylene production by CO_2_RR [119]. Nanoscaled defects, 2–14 nm in size, in Cu(111) nanosheets enhance the absorption and concentration of *CO and *OCCO intermediates as well as with hydroxyl ions which promote C-C coupling. The FE reached 83.2% at a current density of 60 mA cm^−2^ and −1.2 V vs. RHE in 0.1 M K_2_SO4 electrolyte.

The hierarchical construction of a Sn-Cu/SnOx core/shell catalyst with a core of Cu-Sn alloy and metallic Sn and an amorphous SnO2 shell favors its activity and selectivity towards CO and formate [120]. The FE of the Sn_2.7_Cu catalyst to C1 products was 98.0% (−0.70 V vs. RHE) at 406.7 mA cm^−2^ current density. Stability was monitored for 40 h; FE reached 99.0% (−0.55 V vs. RHE) at current density of 243.1 mA cm^−2^. During CO2RR, the Sn in the core diffuses to the shell SnO_2_ reconstructing the Sn/SnO_2_ interface that weakens the *H bonding and favors the formation of the intermediate HCOO* to produce formic acid. DFT calculations detected the reconstruction of Sn/SnOx in the catalyst. Single-atom copper catalysts supported on nitrogen-doped graphene-like structures were synthesized by Rai et al. [121] through reduction of Cu^2+^ ions followed by thermal treatment at 800 °C under an inert atmosphere. This process resulted in well-dispersed copper single atoms at trace levels anchored on the N-doped graphene structure. The presence of copper and nitrogen increased the defect sites on the graphene, thereby enhancing the CO_2_ reduction reaction. High faradaic efficiency was obtained at −0.8 V vs. RHE with values of 91.0%, compared to 59% for ethylene. EXAFS analysis was performed by detecting the reoxidation of the catalysts after CO2RR. Also, Wan et al. synthesized a PrxO/Cu catalyst with high performance for C2+ products by modifying the spatial distribution of both phases [122]. Superficial PrO_x_-coated Cu particles (sPrO_x_/Cu) were obtained by liquid-phase diffusion, while encapsulated PrO_x_ (hPrO_x_/Cu) was prepared by Thermal reduction. sPrO_x_/Cu showed selectivity to ethylene with an FE of 85% (500 mA cm^−2^), but hPrO_x_/Cu FE only reached 72%, with CO FE > 21%. This is because superficial PrO_x_ acts as a co-catalyst in water activation.

The role of Cu(111) and Cu(100) modification during CO_2_RR was theoretically predicted and confirmed by scanning tunneling microscopy [123]. The formation of steps or kinks under CO_2_RR conditions, with adsorbed CO and negative potentials, is key to the activity and selectivity of Cu (111) and (100) planar surfaces. The higher density of these defects improves catalyst performance because they are thermodynamically stable during CO_2_RR. C2+ production depends on CO coverage and the intrinsic coupling barrier; defective Cu(100) offers a lower barrier and higher CO coverage, enhancing C2+ products while simultaneously inhibiting H2 formation. As reported by Peng et al. [98], grain boundaries of Cu-based catalysts can be modulated by an additive (sodium pyridine-2-carboxylate)-assisted electrodeposition method. The materials present grain boundaries in which Cu(110), Cu(111), and Cu(100) coexist. In situ ATRFTIR spectroscopy revealed that grain boundaries enhance CO absorption during C-C coupling and suppress H_2_ production. FE reaches 57.7% for C2+ (−1.08 V vs. RHE) and 43% for C_2_H_4_ in an H cell, while a flow cell yields a maximum C2+ FE of 50.03%.

#### 3.2.5. Overview of Contributing Factors for CO_x_

The electrocatalytic performance of Cu-based materials toward CO_x_RR results from key design factors, including synthesis conditions, Cu oxidation state, alloying and doping, morphology and crystallographic facets, defects, and interface interactions. All these factors together determine the catalyst’s surface and Cu electronic structure, the distribution of active sites, and the adsorption and stabilization of reaction intermediates; their combined effect also determines catalytic activity and selectivity. Consequently, they also govern CO_x_ reduction pathways to C1 and multicarbon products and inhibit HER. Synthesis and post-synthesis treatments provide the first tool for controlling catalyst composition, morphology, exposed facets, defect density, oxidation state, and interfacial structure. In particular, the coexistence of Cu^0^ and Cu^+^ species, together with specific facets, defects, steps, and grain boundaries, can modify *CO adsorption and surface coverage to promote C–C coupling, a key step toward C2+ products. Alloying and doping also provide another way to tune Cu electronic structure and oxidation state, modifying intermediate adsorption and suppressing H2 formation. In situ and ex situ characterization techniques complemented by theoretical calculations are essential for identifying active species, reaction intermediates, and structural transformations during CO_x_RR.

## 4. Cutting Edge Characterization Techniques

### 4.1. In Situ and Operando Insights into Cu-Bimetallic Catalysts for CO_2_ and Nitrate Ion Electroreduction

The electrochemical reduction of nitrate ions and carbon dioxide has emerged as a promising pathway for sustainable nitrogen–carbon cycle management. Nitrate, a compound present in significant concentrations in agricultural runoff and wastewater, poses substantial environmental and health risks. However, its conversion to ammonia (or less contaminant products such as molecular nitrogen) offers a pathway to decentralized fertilizer production and energy storage. On the other hand, induced electrochemical reduction of carbon dioxide provides an alternative to convert atmospheric carbon into commercially valuable compounds such as fuels and/or chemicals. Among all transition metals, copper is unique in its ability to catalyze the reduction of both NO_3_^−^ and CO_2_. However, monometallic Cu exhibits high overpotentials, competing hydrogen evolution, and limited selectivity. The strategic incorporation of a second metal into Cu-based catalysts—creating bimetallic architectures—has emerged as a powerful strategy to overcome these limitations, enabling synergistic electronic, geometric, and interfacial effects that monometallics cannot.

The primary advantage of bimetallic Cu-M catalysts is their capacity to adjust the electronic structure of the active surface, most notably through the modification of the Cu d-band center. In accordance with d-band theory, the position of the d-band center relative to the Fermi level is a determining factor in the strength of adsorbed-surface species interactions. In monometallic Cu, the d-band center is positioned at a considerable distance from the Fermi level (−2.84 eV), leading to the weak binding of key intermediates such as *NO_3_, *NO_2_, and *COOH. In monometallic Cu, the d-band center is positioned at a considerable distance from the Fermi level (−2.84 eV), leading to the weak binding of key intermediates such as *NO_3_, *NO_2_, and *COOH. This phenomenon necessitates the application of substantial overpotentials to drive reactions in turn. Allowing Cu with a second metal has been demonstrated to shift the d-band center closer to the Fermi level, thereby strengthening intermediate adsorption and lowering activation barriers.

Copper bimetallic catalysts have been shown to exhibit synergistic effects arising from a variety of factors, including electronic interactions, ensemble effects, interfacial sites, strain, modified adsorption energies, and complementary catalytic functions. However, the interpretation of these effects is complicated by the dynamic nature of electrochemical interfaces. The surface structure present after catalyst synthesis might differ substantially from the structure present under reaction conditions. This discrepancy can be attributed to the occurrence of various processes, including electrochemical reduction, dissolution-redeposition, atomic migration, oxidation state changes, adsorption, and morphological reconstruction, which can take place during operation. Consequently, the catalytically relevant material should be regarded as a dynamic Cu-M interface under electrochemical conditions, rather than as the static material characterized before redox processes. This consideration renders in situ and operando characterization particularly important. Spectroscopic and microscopic measurements performed under reaction conditions have been shown to provide information about oxidation state, coordination environment, surface adsorbates, intermediate populations, and structural evolution on Cu-bimetallic catalysts. Such information is critical for establishing experimentally supported relationships between catalyst structure, reaction intermediates, and product selectivity.

### 4.2. Cu-Bimetallic Catalyst for CO_2_ Electroreduction

The electrochemical conversion of carbon dioxide involves a series of proton-coupled electron-transfer steps, resulting in the generation of various products, including carbon monoxide, formate, methane, ethylene, ethanol, and others. Copper is atypical in its ability to catalyze C1 and C2+ pathways; however, this versatility is accompanied by relatively poor selectivity. The development of Cu-bimetallic catalysts, therefore, aims to modify the reaction energetics and surface environment so that specific intermediates and pathways become preferentially stabilized.

A concept that has been the focus of significant research is the tandem catalytic mechanism, in which the second metal facilitates the conversion of CO_2_ to CO, while neighboring Cu sites promote further CO reduction and C-C coupling. In the early stage of research, it was determined that interfaces between CO-producing metal and copper could enhance the formation of reduced carbon products, particularly C2 oxygenate species [124,125,126]. Subsequent operando investigations of CuZn nanoparticles demonstrate that changes in Cu/Zn structure and composition correlate with changes in CO_2_-reduction selectivity, providing direct evidence that the bimetallic structure is not merely a static structural descriptor [127]. More recent work has substantially expanded this concept. Luo et al. reviewed Cu-based bimetallic catalysts and emphasized that the traditional tandem model is inadequate in explaining all observed synergistic effects [128]. The observed catalytic behavior can then be attributed to a multitude of factors, including electronic interactions between the two metals, modifications of intermediate adsorption energies, local species concentration at the initial redox-process sequence-mechanism (for example, CO for this case), interfacial electric fields, and dynamic restructuring. Consequently, a Cu-M interface (and, in general, any (multi)metallic interface for a variety of redox processes) should be regarded as a multifunctional reaction environment rather than merely the juxtaposition-association of two (or more) independent catalytic sites. In this context, in situ RAMAN spectroscopy has emerged as a highly effective technique for investigating these phenomena. RAMAN measurements have been demonstrated to monitor Cu-oxide species, adsorbed CO, and alterations in the surface structure during potential cycling and steady-state redox processes. Recent studies have shown that copper surfaces undergo substantial potential-dependent variations, and that the amount and configuration of adsorbed CO are strongly dependent on the local surface structure [129,130]. These measurements are of value because the formation of *CO is closely associated with the subsequent C-C coupling chemistry, responsible for C_2_+ products.

On the other hand, the significance of catalyst reconstruction has become increasingly evident through recent operando studies. Kim et al. conducted a comprehensive investigation of Cu-X bimetallic electrocatalysts, where X denotes Ag, Fe, Zn or Pd, during the reduction of CO_2_. Utilizing in situ electrochemical liquid-cell transmission electron microscopy in conjunction with complementary structural and computational methods, the authors demonstrate that Cu-bimetallic catalysts undergo substantial surface reconstruction during the redox process in turn. The reconstruction process was associated with selective dissolution-redeposition mechanisms and depended on the miscibility and oxophilicity of the second metal. Of particular significance is the observation that the reconstructed surfaces exerted a notable influence on the CO_2_ reduction and selectivity process, encompassing the equilibrium between C_1_ products and C_2_ products, as well as the distinction between ethylene and ethanol. This study is of particular significance because it demonstrates that the original Cu-M structure cannot necessarily be assumed to represent the catalytic surface. Instead, the reaction intermediates themselves can influence which elements migrate, dissolve, or redeposit. As a result, the relationship between catalyst structure and selectivity can be dynamic and bi-directional: the catalyst controls intermediate formation, while the intermediates simultaneously modify the catalyst—Figure 4. The dynamic behavior of copper is also supported by studies using mono-metallic Cu. Vavra et al. demonstrated that Cu reconstruction during CO_2_-reduction involves soluble Cu(1)-containing species and a dissolution-redeposition mechanism; for this case, online mass spectroscopy and UV-vis spectroscopy provide evidence for transient Cu-species, while theoretical calculations suggest that Cu-adsorbates, such as copper carbonyl and oxalate species, can participate in the reconstruction process [131]. These observations are particularly relevant for Cu-bimetallic systems, as the second metal can influence both the thermodynamics of Cu dissolution and the stability of the resulting surface. It is also essential to consider the role of the electrolyte and the interfacial environment. Recent in situ spectroscopic investigations indicate that interfacial water can participate directly in CO_2_-to-C_2_ conversion by influencing proton transfer, hydrogen-bonding networks, and intermediate stabilization. Therefore, the catalytic effect of a second metal might extend beyond modification of the Cu-electronic structure to include changes in interfacial water structure, local electric fields, and hydrogen availability [132].

### 4.3. Cu-Bimetallic Catalyst for Nitrate-Ion Electroreduction

The electrochemical reduction of nitrate ions offers an alternative pathway for converting an environmentally problematic nitrogen oxyanion into ammonia or other useful nitrogen products. In contrast to the CO_2_RR process, nitrate-reduction reaction (NO_3_RR) involves a series of reductions and hydrogenation steps on nitrogen–oxygen intermediates. Depending on the structure of the catalyst and the operating conditions, nitrate can be converted to NO_2_^−^, NH_3_/NH_4_^+^, N_2_, or other products. Consequently, controlling the adsorption and conversion of intermediate species such as *NO_3_, *NO_2_ and *NO is imperative to ensure high selectivity. The inherent complexity of the reaction network renders in situ an operando characterization of particular significance. Wang et al. provided a comprehensive assessment of in situ/operando methods for nitrate reduction and emphasized that conventional ex situ characterization cannot fully resolve the time-dependent evolution of active sites and reaction intermediates [133]. Their review underscores the significance of Raman, infrared spectroscopy, X-ray absorption spectroscopy, X-ray photoelectron spectroscopy, electrochemical mass spectrometry, and related techniques as complementary approaches for identifying the reaction mechanism under operating conditions.

In the context of Cu-bimetallic systems, the incorporation of a second metal can yield complementary functionality. The second metal has been shown to participate in nitrate adsorption and activation. In addition, it has been demonstrated to modify the adsorption energy of nitrogen-containing intermediates, facilitate water dissociation, provide reactive hydrogen, or alter the electronic structure of neighboring Cu atoms. This multifunctional behavior provides a conceptual basis for Cu–Ni, Cu–Pd, Cu–Fe, Cu–Co, and other bimetallic systems. The combination of Cu and Ni exhibits considerable promise due to the unique properties of these elements. Nickel facilitates hydrogen adsorption and water activation, while Cu facilitates favorable interactions with nitrate-derived species. Recent Cu–Ni catalysts have exhibited high activity toward nitrate-to-ammonia conversion, thereby supporting the hypothesis that different elementary steps can be distributed between neighboring Cu and Ni sites [134]. It is important to note that the Cu/Ni ratio and the local coordination environment exert a significant influence on the binding of nitrogen-containing intermediates. This, in turn, affects the competition between further reduction and NO_2_^−^ release. The low-coordination Cu–Ni sites offer a particularly interesting illustration of geometric control. Recent studies of Cu–Ni aerogels have demonstrated that low-coordinated Cu–Ni edge sites can enhance nitrate-to-ammonia conversion by modifying the electronic structure and adsorption characteristics of nitrate-derived intermediates [135]. These results underline the necessity of considering additional factors to adequately describe catalytic behavior. Specifically, the coordination number, atomic arrangement, and local Cu–Ni environment must be considered when evaluating the elemental composition. Cu–Pd systems provide another important example. Recent studies of ordered CuPd nanocrystals have demonstrated that the atomic ordering of the intermetallic phase can strongly influence nitrate reduction to ammonia [136]. Ordered B2-CuPd and disordered CuPd structures provide different surface ensembles and therefore different adsorption environments for nitrate-derived intermediates. These observations reinforce the importance of distinguishing between a homogeneous alloy (an ordered intermetallic compound) with respect to a heterogeneous Cu/Pd interface.

### 4.4. Dynamic Oxidation States and Reconstruction During NO_3_RR

A recent trend in the field of nitrate electroreduction suggests that the oxidation state of copper (Cu) can undergo significant alterations during the redox process. This situation is of particular importance because Cu(I), Cu(II), and Cu(0) can exhibit substantially different adsorption characteristics and catalytic properties. In this context, in situ studies have demonstrated that Cu-based precursors can undergo electrochemical reconstruction to generate mixed-valence Cu configurations during nitrate reduction [39]. In situ Raman and XPS measurements, complemented with other in situ techniques, have provided evidence that Cu^1+^/Cu^0^ and Cu^2+^/Cu^0^ configurations can develop under working conditions. These mixed-valence configurations might alter nitrate activation, hydrogen generation, and the subsequent hydrogenation of nitrogen-containing intermediates. Then, this observation is conceptually important for Cu-bimetallic catalysts due to the fact that the second metal may influence the potential at which Cu changes oxidation states and the stability of particular Cu^0^/Cu^+^ or Cu^+^/Cu^2+^ redox-couple configurations: bimetallic catalysis can involve not only Cu–M atomic ensembles but also potential-dependent oxidation-states [39]. The implication is that the catalytically relevant descriptor may not be simply “Cu–M alloy composition.” Instead, a more complete descriptor should consider: *Cu/M composition + atomic coordination + oxidation state + adsorbate coverage + interfacial environment* [137]. In this context, in situ and operando techniques are essential for distinguishing between the initial catalyst and the working catalyst [138,139,140]; see Figure 4.

### 4.5. Toward a Unified Description of Cu–M Interfaces

Despite the fundamentally different chemistry of CO_2_RR and NO_3_RR, a common framework can be developed for Cu-bimetallic electrocatalysis. In both reactions, the second metal has the capacity to modify the Cu surface through electronic, geometric, and interfacial effects. More precisely, the Cu–M structure can evolve during the redox process, meaning that catalytic activity and selectivity might depend on a continuously changing population of active sites, as observed from in situ techniques.

(a) For carbon dioxide reduction, a simplified pathway can be represented as follows

CO_2_ → COOH → CO → C–C coupling → C_2_ + products

where the formation, accumulation, and transformation of CO are central to multi-carbon products.

(b) For NO_3_RR, a simplified pathway can be expressed as

*NO_3_ → NO_3_^−^ → NO_2_^−^ → NO → hydrogenated intermediates → NH_3_/NH_4_^+^ or N_2_.

In both cases, the presence of Cu–M interfaces has been demonstrated to stabilize various intermediates and facilitate the progression through sequential elementary steps. This provides a mechanistic basis for designing tandem or multifunctional catalytic interfaces.

### 4.6. Perspectives

In summary, the recent literature points toward a fundamental shift in the design philosophy of Cu-bimetallic electrocatalysts. The comparison of CO_2_RR and NO_3_RR suggests that, despite their different reaction chemistries, both can be interpreted through a common structure–active-site–performance framework in which electronic structure, atomic coordination, oxidation or valence state, morphology, exposed facets, local reaction environment electron donors, and interfacial effects govern the stabilization and transformation of key intermediates. For this reason, controlling selectivity is important because the interplay among NO_3_^−^ reduction, CO_2_ reduction, hydrogen evolution, and C–N coupling is characterized by a delicate equilibrium. Precise control over bimetallic or multimetallic composition, morphology, and support interactions is imperative to regulate selectivity.

Rather than optimizing only the composition and morphology of the as-synthesized catalyst, future studies should seek to control the dynamic operando Cu–M interface, since the catalytically relevant surface can differ substantially from the initial material as a consequence of reconstruction, changes in oxidation state, atomic migration, adsorbate coverage, and changes in the interfacial environment. Additionally, long-term stability should be considered: bimetallic catalysts, particularly those containing Ni, may undergo metal leaching or phase segregation during extended electrolysis. Implementing protective supports and surface-engineering strategies is imperative.

The most relevant parameters therefore include the distribution of Cu and M atoms, the oxidation state of copper, the coordination number, the defect density, the coverage of adsorbates, the interfacial water structure, and the local concentration of key reaction intermediates. Consequently, in situ and operando characterization should not be regarded merely as a supplementary characterization step, but rather as a central component of catalyst design. The integration of operando attenuated total reflection infrared and Raman spectroscopy, microscopy, electrochemical mass spectrometry, isotope labeling, kinetic analysis, and theoretical modeling such as density functional theory (DFT) calculations facilitates the establishment of direct connections between the dynamic Cu–M interface, key reaction intermediates, and product selectivity.

From this perspective, catalyst development can be optimized considering mechanistically informed design rules. Cu–M interfaces may be engineered as multifunctional environments in which neighboring sites perform complementary elementary steps, stabilize distinct intermediates, control local reactant or hydrogen availability, and suppress competing pathways such as HER. In CO_2_RR, this includes controlling CO formation, surface coverage, and C–C coupling toward C_2_+ products, whereas in NO_3_RR it involves coordinating nitrate activation and the sequential hydrogenation of nitrogen-containing intermediates. Thus, a central perspective emerging from both reaction families is that future Cu-based nanocatalysts should be designed not only for a target composition or morphology, but for a target operando interfacial state and reaction sequence. Coupling catalysts for a co-reduction process is a promising yet underexplored avenue for directly generating carbon–nitrogen compounds. For example, the study of C–N synthesis throughout the Cu–M system, with strong adsorption of NO_x_ at M sites and copper activating CO_2_, is a particularly salient area of research.

Furthermore, regarding scale-up and device integration, the majority of in situ studies use model systems, which may include thin films, single crystals, or dilute loadings. The translation of these insights into high-current-density flow cells and membrane electrode assemblies (MEA_s_) remains an engineering challenge.

Finally, future research must prioritize the following areas: (i) the implementation of machine-learning algorithms to facilitate the screening of bimetallic compositions and supports; (ii) the development of time-resolved operando techniques to enable the capture of transient intermediate states; (iii) the utilization of isotope-labeling MS to ensure the unambiguous tracing of product genesis in co-reduction systems; and (iv) the execution of industrial-relevant testing in flow electrolyzers utilizing real wastewater and flue gas feeds.

## 5. Conclusions

This work reviews the literature on preparing and structurally regulating Cu-based nanomaterials as catalysts for the electroreduction of NO_x_ and CO_x_ for pollution control and the production of commercially relevant products. By comparing both reaction families, the review identifies common catalyst-design variables and reaction-specific requirements linking nanomaterial structure, active sites, reaction intermediates, and catalytic performance. The review highlights the relevance of cutting-edge in situ and operando characterization techniques.

- Catalyst preparation strategies capable of generating Cu-based nanomaterials are particularly advantageous as they determine the structural properties that, in turn, determine the distribution, nature, and density of active sites and the adsorption and stabilization of reaction intermediates in both NO_x_ and CO_x_ electroreduction. Catalyst performance should be understood as a combination of these descriptors.
- Cu-based nanomaterials have shown high activity for NO_x_ and CO_x_ electroreduction reactions; however, new concepts are needed to move to practical implementations. In this context, recent approaches increasingly extend toward interfacial microenvironment engineering, including local pH, interfacial water structure, reactant enrichment, and proton availability.
- In situ and operando techniques such as DEMS, RAMAN, EXAFS, and IRAS coupled with electrochemical techniques successfully contribute to elucidate reaction sequences and, after appropriate calibration, the selectivity and conversion of the catalyst in turn. Recent approaches combining theoretical calculations, time-resolved operando characterization and atomically defined Cu–M architectures provide a route to correlate catalyst reconstruction with intermediate coverage, and product selectivity. Such strategies may shift catalyst design from static structural descriptors to experimentally validated operando descriptors.

Therefore, a central conclusion from comparing NO_x_ and CO_x_ electroreduction is that both reactions can be interpreted through a common structure–active-site–performance framework, while retaining reaction-specific requirements for intermediate stabilization and product formation. For practical implementation, this design philosophy should be evaluated rigorously under technologically relevant conditions using gas-diffusion electrodes, flow cells, and membrane electrode assemblies, where mass transport, current density, energy efficiency, and long-term durability become as important as intrinsic catalytic activity. The recent literature on both CO_2_RR and NO_3_RR increasingly treats these device-level metrics, together with techno-economic and life-cycle considerations, as necessary for assessing industrial relevance.

## Figures and Tables

**Figure 1:**
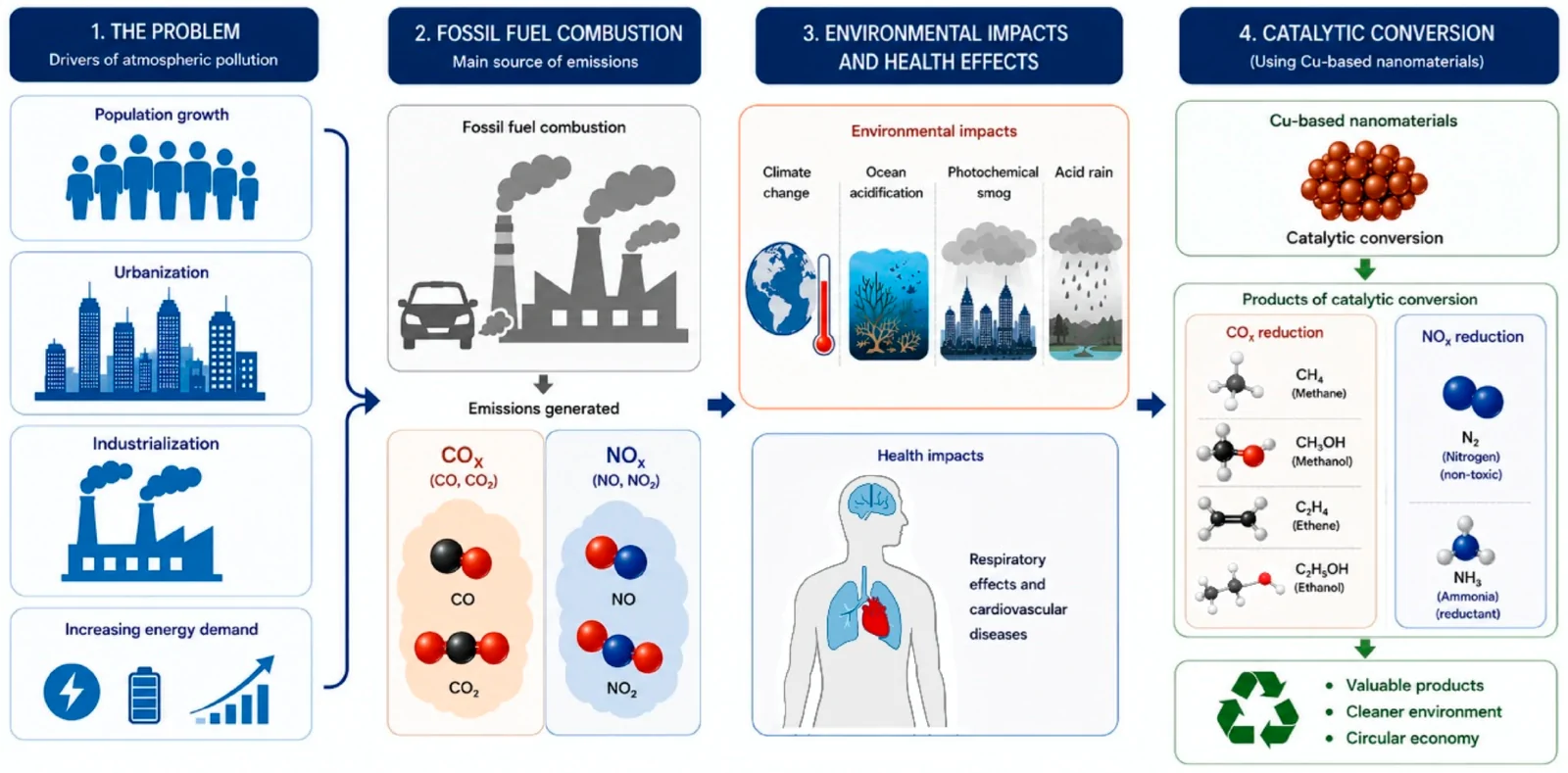
Environmental context and electrochemical conversion of CO_x_ and NO_x_ using Cu-based nanomaterials. The diagram summarizes anthropogenic drivers of fossil fuel combustion, the resulting emissions, and their environmental and health impacts. It also illustrates conversion pathways toward carbon products such as CH_4_, CH_3_OH, C_2_H_4_, and C_2_H_5_OH, and nitrogen products such as N_2_ and NH_3_.

**Figure 2:**
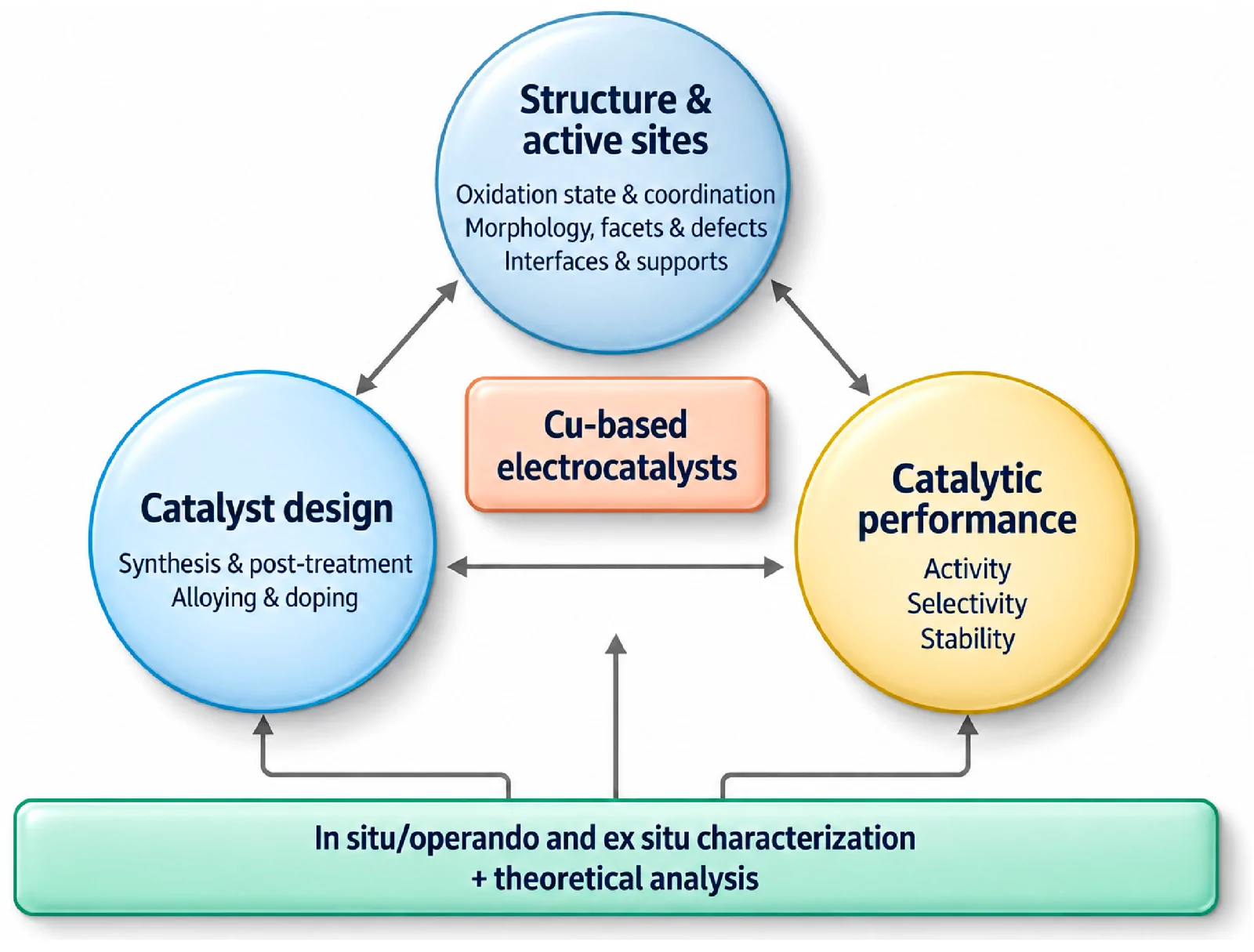
Conceptual framework linking catalyst design, structure and active sites, and electrocatalytic performance in Cu-based materials. Bidirectional arrows represent the interdependence of these aspects, while characterization and theoretical analysis provide evidence to interpret their relationships and guide catalyst development.

**Figure 3:**
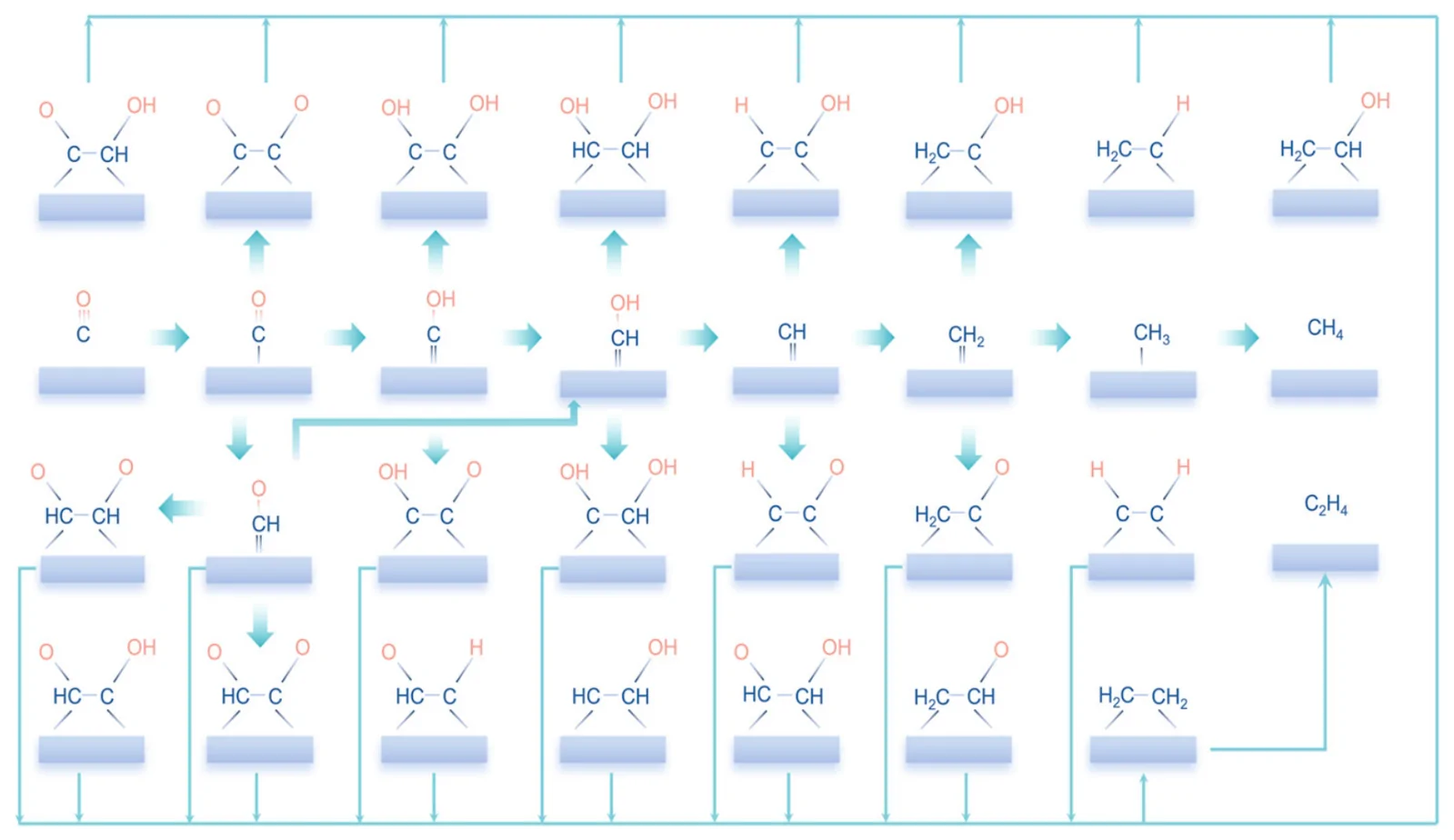
Representation of intermediates for C1 and C-C coupling. Reproduced with authorization of RSC from [97].

**Figure 4:**
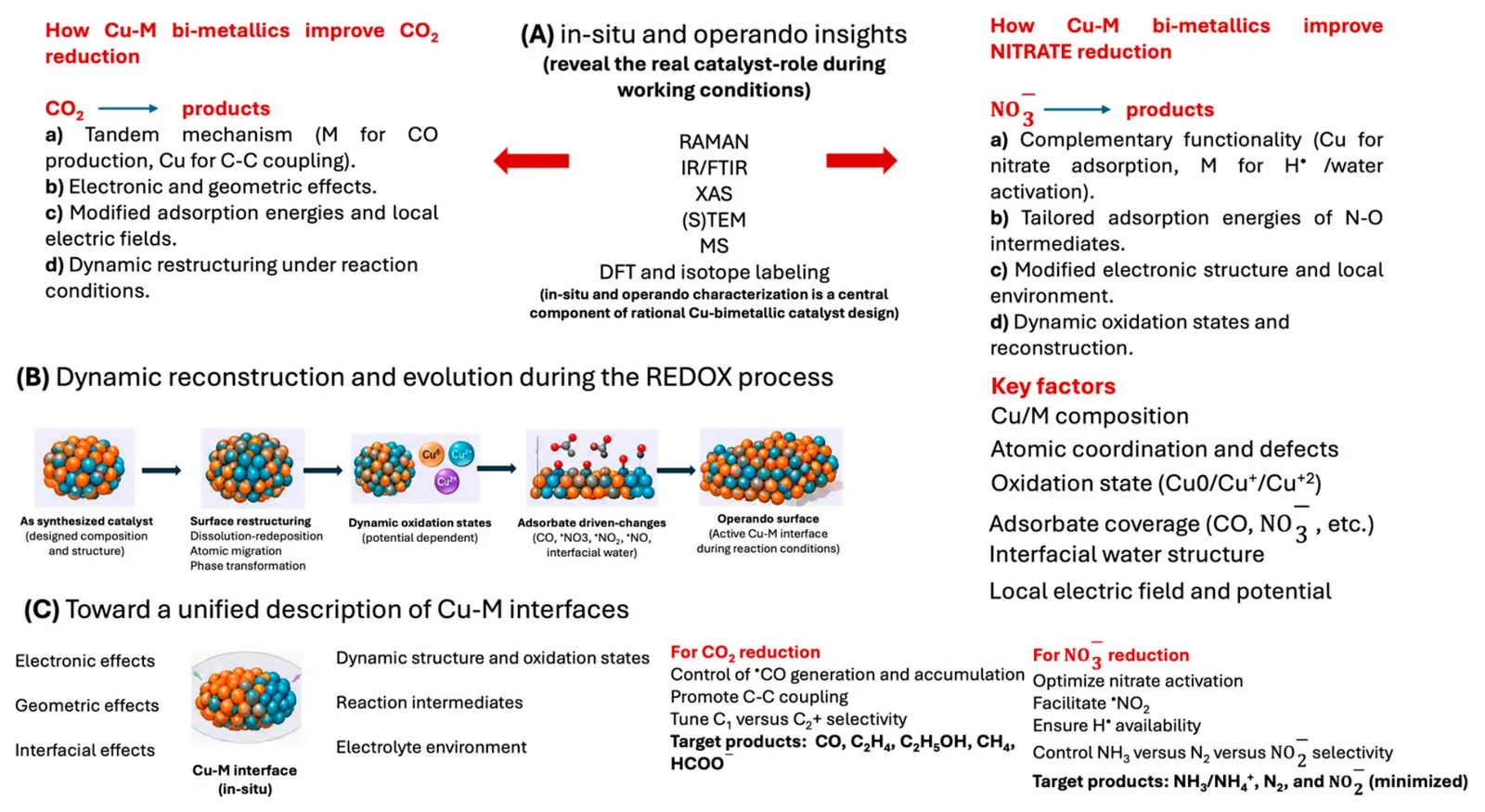
Operando techniques and their impact on the design of bimetallic catalysts for induced REDOX reactions: nitrate reduction and carbon dioxide reduction.

**Table 1:** Summarizes Cu-based materials employed in NO_x_ electrochemical reduction reaction.

Catalyst	Morphology	Particle Size [nm]	Chemical Species	Current Density [mA cm^−2^]	Selectivity	FE [%]	Cutting Edge Characterization Approaches	Ref.
Cu_2_O	Cubic	30–40	NaNO_3_ 8 mM	−7	NH_3_	80	In situ RAMAN, Quasi in situ XPS, XAS	[22]
			NaNO_2_ 8 mM	−6		90		
CuO	Assembled networks		KNO_3_ 0.1 M		NH_3_	95.72	In situ RAMAN, XPS, in situ FIT-IR, DEMS, HRTEM	[39]
Cu_2_P_2_O_7_						94.88		
Cu_3_(PO_4_)_2_						92.04		
CuFe-300	Nanosheet						HRTEM, XPS, In situ FIT-IR, HRTEM	[40]
CuFe-400								
CuFe-450		25	NO_3_^−^ 3 mM		NH_3_	90.6		
CuFe-450						87.1		
PdCu	Semi-spherical	3	NO_3_^−^ 0.01 M	−0.25			XPS, DEMS	[33]
			NO_3_^−^ 0.1 M	−0.27				
			NO_3_^−^ 1 M	−0.34				
			NO_2_^−^ 0.1 M	−0.87				
			NO_2_^−^ 1 M	−3.7				
Cu/GP*	Multiple layers of GP	45.8					RAMAN, XPS	[41]
Cu/rGO*/GP*		28.5	NO_3_^−^ 0.002 to 0.02		NH_3_	29.93		
Cu/Ni[foam]	Granule, rod, rhombus and hexagon	20–35	NO_3_^−^ 0.003 M		N_2_	55.6	XPS	[42]
			NO_3_^−^ 0.006 M					
			NO_3_^−^ 0.01 M					
			NO_3_^−^ 0.015 M					
Cu-in-CNT*		~8.7	KNO_3_ 0.1 M		NH_3_	70	XPS, ICP-OES, ADF-STEM	[43]
Cu-on-CNT*		~9.5				65		
Cu nanosheets	Triangular		KNO_3_ 10 mM		NH_3_	99.7	AFM, HRTEM	[44]
Pt-Cu NRs*	Nanorods		KNO_3_ 0.1 M	−8.7 × 10^−1^	NH_3_		FESEM, HRTEM, XPS, HAADF-STEM	[45]
Copper single crystals (111), (100)			NaNO_3_ 2 mM	−2.5 (111)			In situ mass spectrometry, in situ ion chromatography, in situ Fourier transformed infra-red spectroscopy	[46]
				−3.9 (100)	H_3_NO (100)			
			NaNO_2_ 10 mM	−4.1 (111)				
				−4.5 (100)				

Note: GP* = Graphite plate, rGO* = Reduced graphene oxide, CNT* = Carbon nanotubes, NRs* = Nanorods.

**Table 2:** Cu-based electrocatalyst for CO_x_ electrochemical reduction reaction.

	Morphology	Particle Size [nm]	Chemical Species	Current Density [mA cm^−2^]	Selectivity	FE [%]	Cutting Edge Characterization Approaches	Ref.
Cu			KHCO_3_ 0.1, 0.5 M		C_2_H_4_ + C_2_H_6_	19.2, 11.2	FE-SEM	[78]
Cu_2_O_OE*_			KHCO_3_ 0.1, 0.5 M		C_2_H_4_ + C_2_H_6_	24.8, 25.6	FE-SEM	[78]
Cu/VC*		4–25	KHCO_3_ 0.1 M		CH_4_	22	XPS	[79]
Cu/SWNT*		10–25	KHCO_3_ 0.1 M		CH_4_	18	XPS	[79]
Cu/RGO*		25–90	KHCO_3_ 0.1 M		CH_4_	20	XPS	[79]
Cu/OLC*		10–30	KHCO_3_ 0.1 M		C_2_H_4_	60	XPS	[79]
Cu_2_O			NaHCO_3_ 0.5 M	−54_(geo)_	CH_3_CHO	5.4	XPS	[74]
CuBi_2_O_4_			NaHCO_3_ 0.5 M	−55_(geo)_	HCOO^−^		XPS	[74]
CuBi_2_O_4_			NaHCO_3_ 0.5 M	−48_(geo)_	HCOO^−^		XPS	[74]
CuBi_2_O_4_			NHCO_3_ 0.5 M	−37_(geo)_			XPS	[74]
SnO_2_-CuO@AC*		5.38	KHCO_3_ 0.1 M	−7.64	HCOOH	78.8	FE-SEM, XPS	[80]
Cu-CrO_2_			KHCO_3_ 0.1 M		C_2_H_4_ + C_2_H_5_OH	78.5	FE-SEM, BET, XPS	[81]
Cu_2_O (111)NPs	Octahedral		KHCO_3_ 1M	−2.5	C_2_H_4_	41.2	SAED, In situ ATR-SEIRAS	[82]
Cu	Spheric	6	KHCO_3_ 0.1 M		C_2_H_4_	20		[83]
Cu	Cubes	44	KHCO_3_ 0.1 M		C_2_H_4_	57		[83]
Cu	Octahedral	150	KHCO_3_ 0.1 M		CH_4_	51		[83]
Cu		50–60	KHCO_3_ 0.1 M		C_2_H_4_	41.9	FE-SEM, FT-IR	[84]
CuO	Rounded	11.4	KHCO_3_ 0.5 M		HCOO^−^	66		[85]

Note: OE* = Oxygen-evacuated, VC* = Vulcan Carbon, SWNT* = Single-wall carbon nanotubes, RGO* = Reduced graphene oxide, OLC* = Onion-like carbon, AC* = Activated carbon.

## Data Availability

The original contributions presented in this study are included in the article. Further inquiries can be directed to the corresponding authors.
